# Supplementary material for: Latent Space Phenotyping: Automatic Image-Based Phenotyping for Treatment Studies
Source: Plant Phenomics. 2020 Jan 20;2020:5801869. doi: 10.34133/2020/5801869 (PMC7706325; doi:10.34133/2020/5801869)
Supplement: Supplementary Materials — Figure S1: the deep network used in learning an embedding. A CNN takes a sequence of images at various time points and feeds outputs to an LSTM, which in turn is used to predict the treatment. The LSTM is removed and the CNN is retained to embed new samples. Figure S2: real images from the Setaria, synthetic Arabidopsis, and sorghum datasets (left) and the same images predicted from their latent space encodings by a decoder network (right). Figure S3: additional examples of synthetic Arabidopsis rosettes (left) decoded from their latent space vectors (right). Table S1: architecture details for the convolutional neural network used in the embedding. All pooling layers are followed by batch normalization. Table S2: architecture details for the decoder network. The upsample blocks refer to transposed convolutions. Figure S4: distance between embeddings of images of lines at different angles in a hypothetical latent space. Here, the semantic distance is the difference in the interior angle. The Euclidean distance between images in the image space is constant, and the Euclidean distance (dotted arrow) between their encodings in the latent space is evidently not representative of the semantic distance. However, the geodesic path (solid arrow) between images represents the semantic distance well. Figure S5: untreated (left) and treated nonresistant (right) synthetic Arabidopsis plants at the final time point, showing differences in leaf elevation angle. Figure S6: ablation experiment using Euclidean image distance between each pair of images in the sequence for the synthetic Arabidopsis dataset. The naive solution fails to recover the simulated tolerance QTL on chromosome 1. Figure S7: well-watered (left) and water-limited (right) examples of a particular line from the Setaria RIL population [12]. Figure S8: well-watered (left) and water-limited (right) examples of a particular line from the B. napus L. NAM population. Figure S9: example images from the Setaria, synthetic [file 5801869.f1.docx]

Supplementary Materials

Predicted


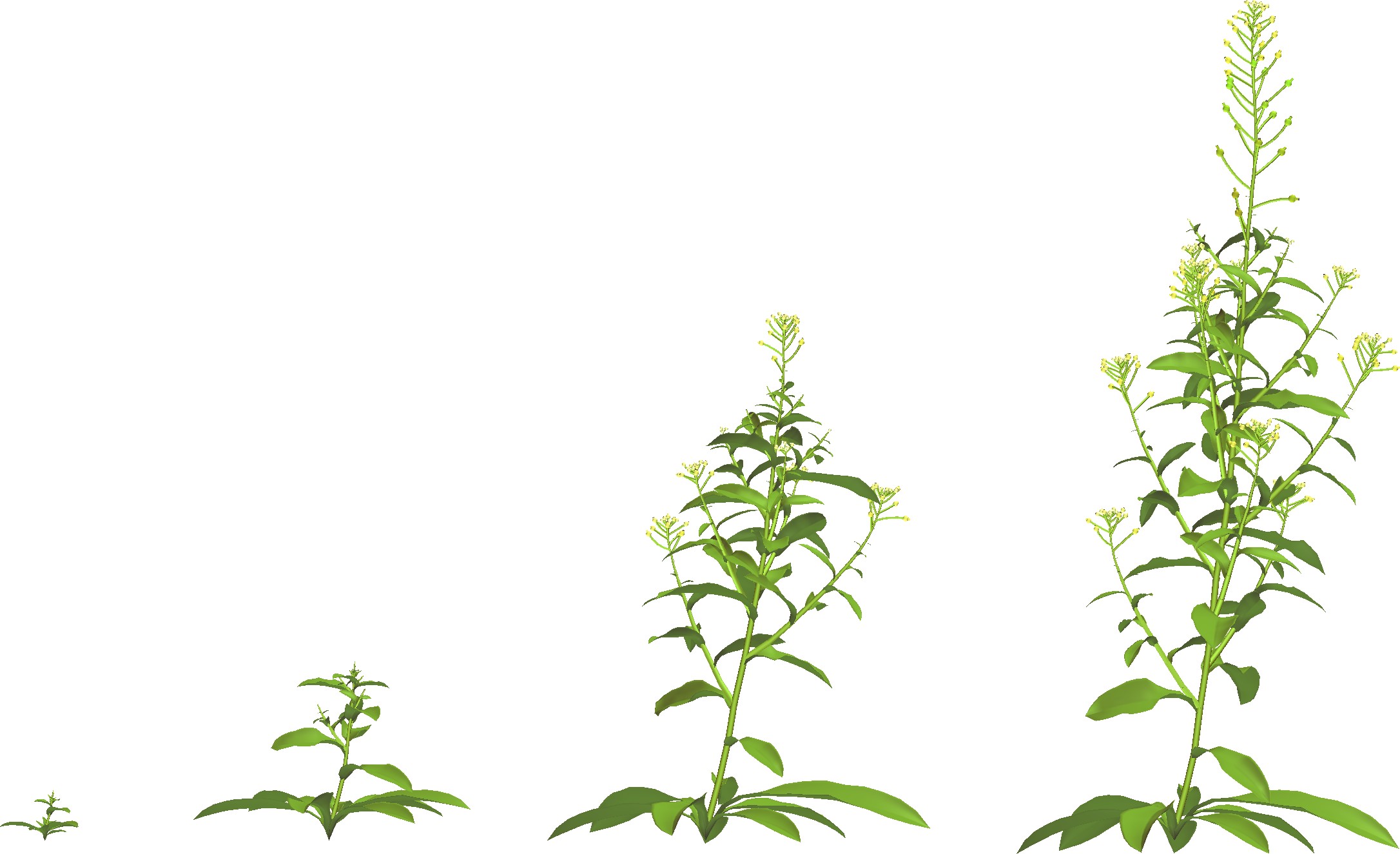

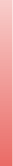

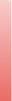

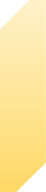

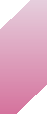

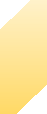

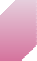

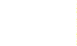

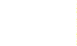

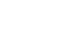

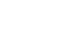

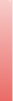


Time

n

n

n

n

LSTM

LSTM

LSTM

LSTM


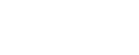

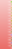


Loss

Treatment

Treatment

Figure S1: The deep network used in learning an embedding. A CNN takes a sequence of images at various timepoints and feeds outputs to an LSTM, which in turn is used to predict the treatment. The LSTM is removed and the CNN is retained to embed new samples.


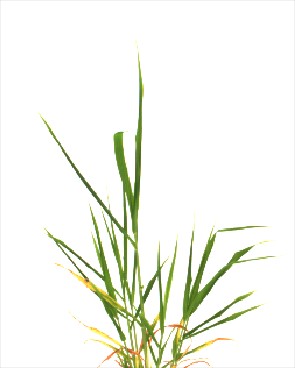

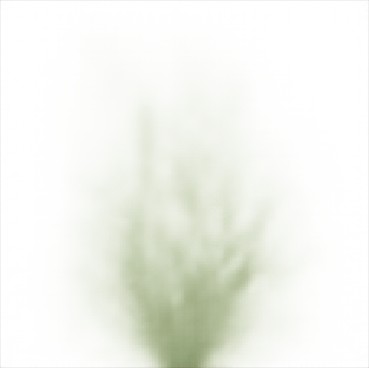


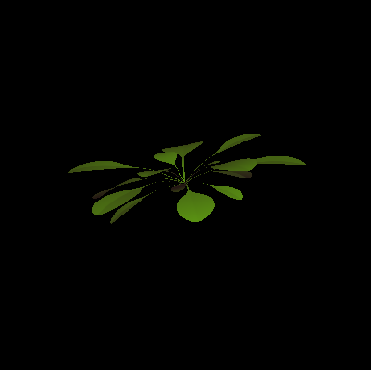

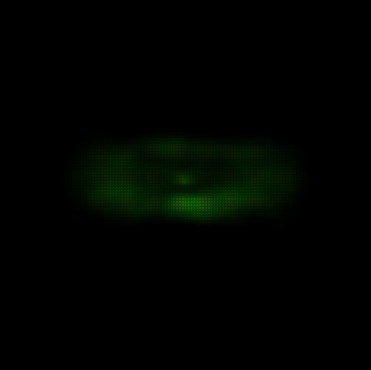

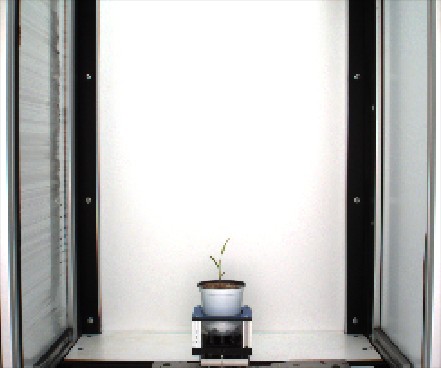

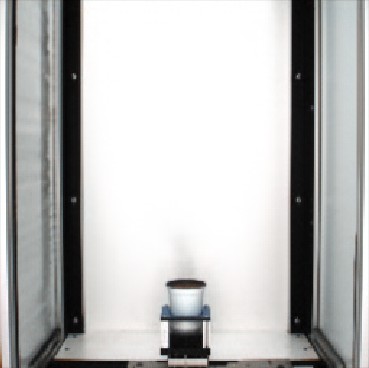


Figure S2: Real images from the *Setaria*, synthetic Arabidopsis, and sorghum datasets (left), and the same images predicted from their latent space encodings by a decoder network (right).


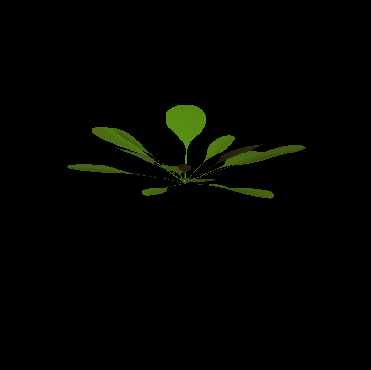

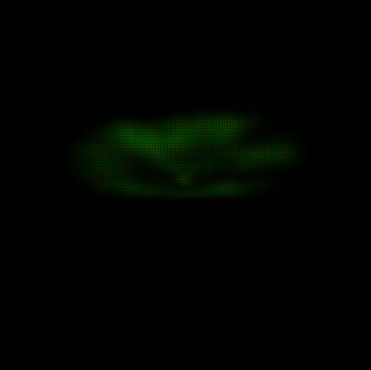


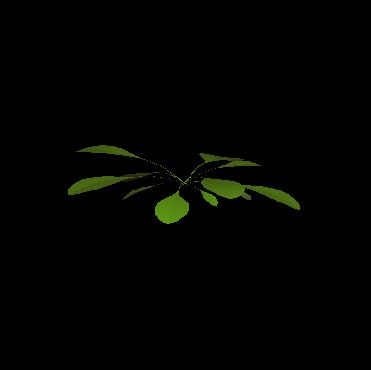

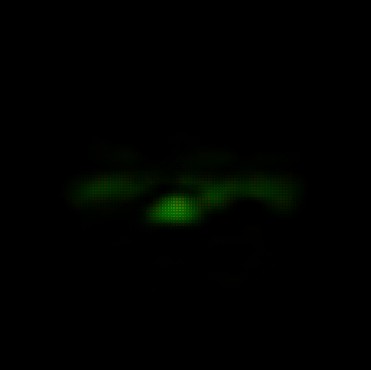


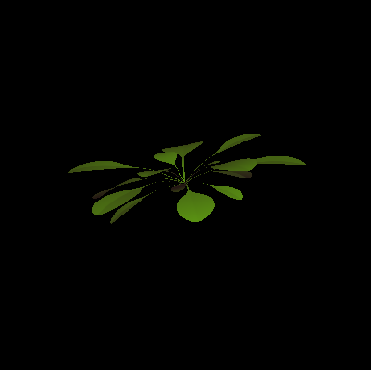

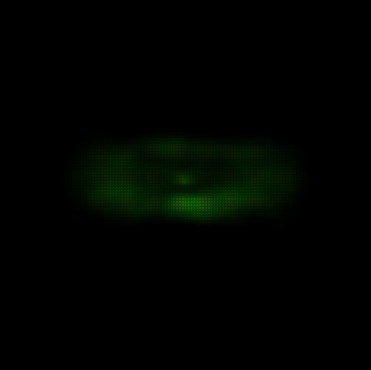


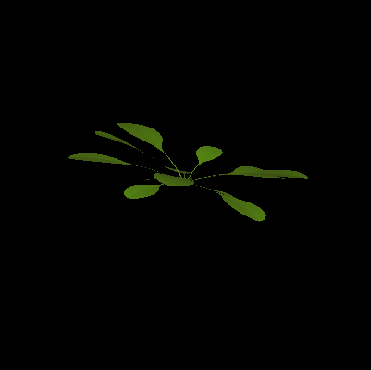

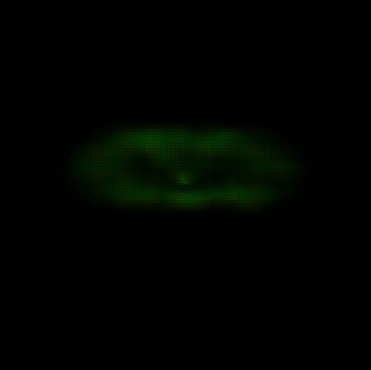


Figure S3: Additional examples of synthetic Arabidopsis rosettes (left) decoded from their latent space vectors (right).

max pool 3 × 3

| Size | Depth | Stride |
| --- | --- | --- |
| conv + relu 3 × 3 | 1 | 1  3 |
| conv + relu 3 × 3 | 16 | 1 |
| max pool 3 × 3 | 32 | 3  1 |
| max pool 3 × 3 | 32 | 3  1 |

conv + relu 3 × 3

conv + relu 3 3

×

max pool 3 3 2

×

fully connected + relu 64

fully connected 16

Table S1: Architecture details for the convolutional neural network used in the embedding. All pooling layers are followed by batch normalization.

Size Depth Stride

conv + relu 3 × 3 16 1

upsample 3 × 3 2

conv + relu 3 × 3 32 1

conv + relu 3 × 3 32 1

conv + relu 3 × 3 32 1

upsample 3 × 3 2

conv + relu 3 × 3 32 1

conv + relu 3 × 3 32 1

conv + relu 3 × 3 32 1

upsample 3 × 3 2

conv + relu 3 × 3 64 1

conv + relu 3 × 3 64 1

conv + relu 3 × 3 64 1

upsample 3 × 3 2

conv + relu 3 × 3 64 1

conv + relu 3 × 3 64 1

conv + relu 3 × 3 64 1

upsample 3 × 3 2

conv + relu 3 × 3 32 1

conv + relu 3 × 3 32 1

conv + relu 3 × 3 16 1

upsample 3 × 3 2

conv + relu 3 × 3 16 1

upsample 3 × 3 2

conv 1 × 1 16 1

Table S2: Architecture details for the decoder network. The upsample blocks refer to transposed convolutions.

Geodesic distance Euclidean distance

90°


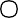

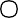

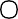

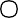

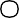

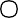


20°

76°

34°

62°

48°

Figure S4: Distance between embeddings of images of lines at diﬀerent angles in a hypo- thetical latent space. Here, the *semantic distance* is the diﬀerence in the interior angle. The euclidean distance between images in the image space is constant, and the euclidean distance (dotted arrow) between their encodings in the latent space is evidently not representative of the semantic distance. However, the geodesic path (solid arrow) between images represents the semantic distance well.


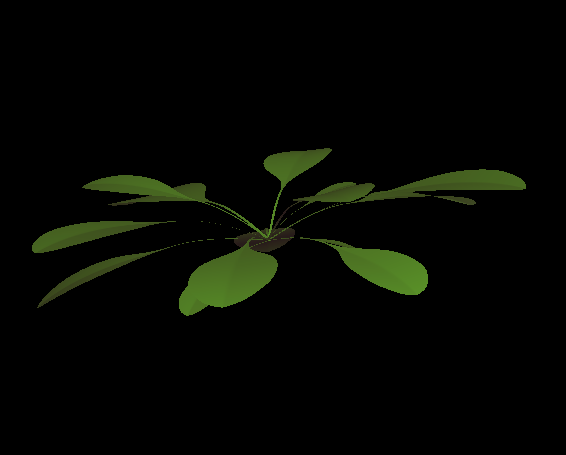

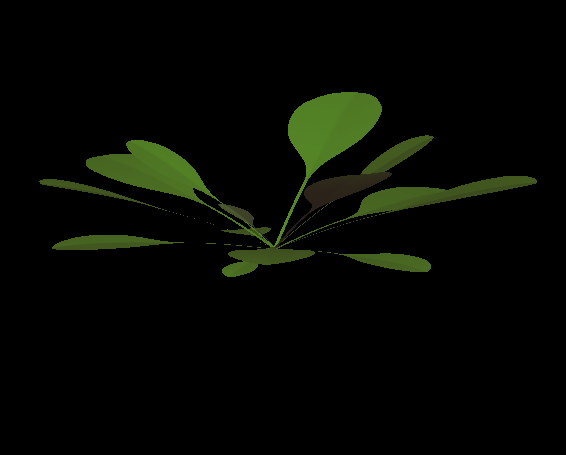


Figure S5: Untreated (left) and treated non-resistant (right) synthetic Arabidopsis plants at the final timepoint, showing diﬀerences in leaf elevation angle.


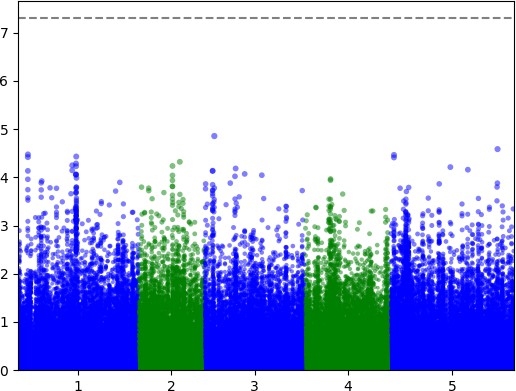


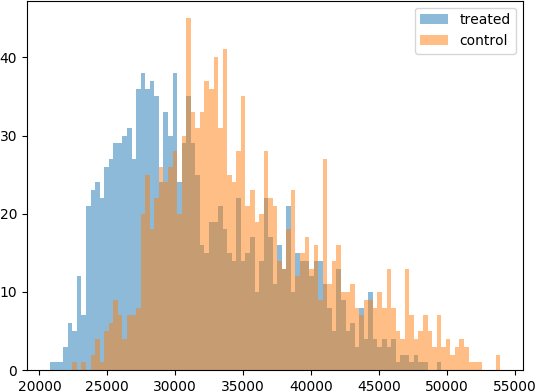

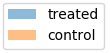


treated

control

Trait Value Chromosome

-log10 (P value)

Figure S6: Ablation experiment using euclidean image distance between each pair of images in the sequence for the synthetic Arabidopsis dataset. The naive solution fails to recover the simulated tolerance QTL on chromosome 1.


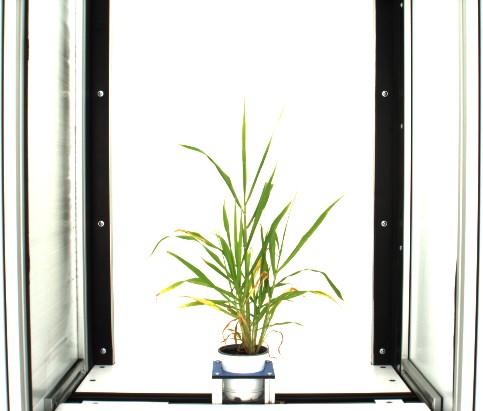

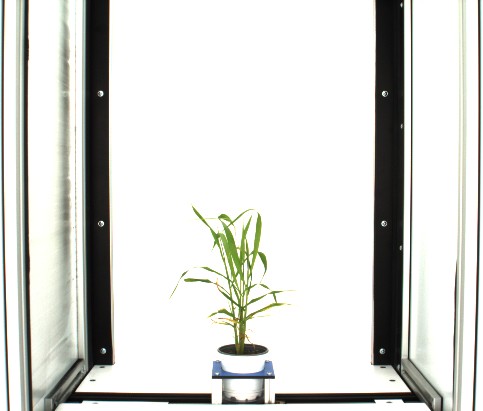


Figure S7: Well-watered (left) and water-limited (right) examples of a particular line from the *Setaria* RIL population [[12](#_bookmark0)]


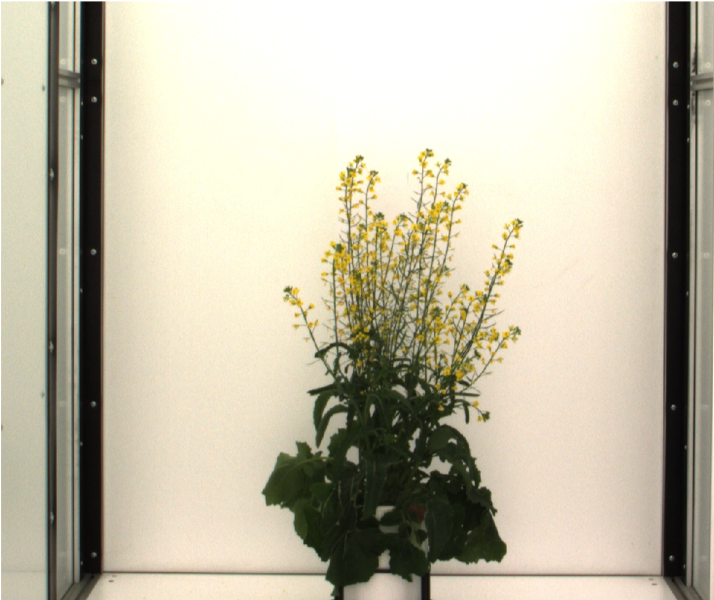

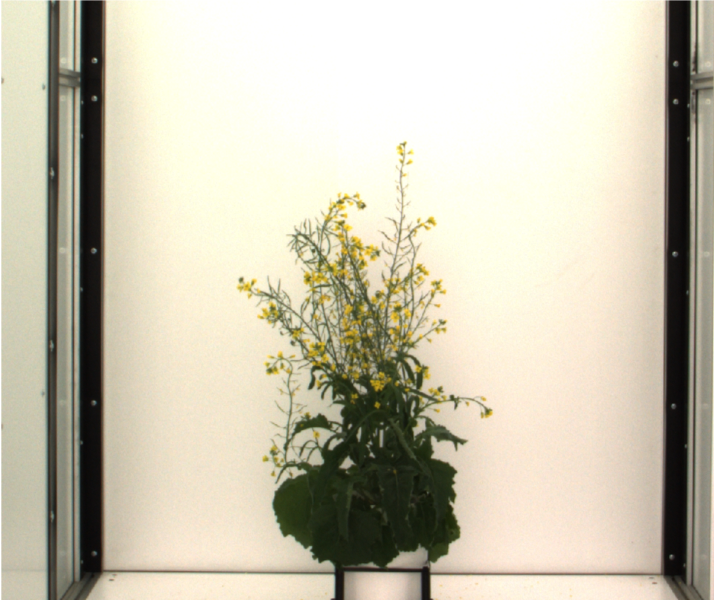


Figure S8: Well-watered (left) and water-limited (right) examples of a particular line from the *B. napus L.* NAM population


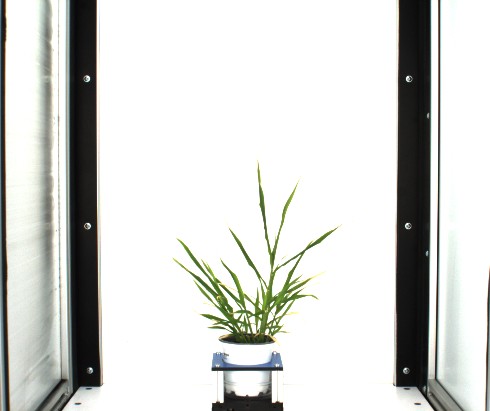

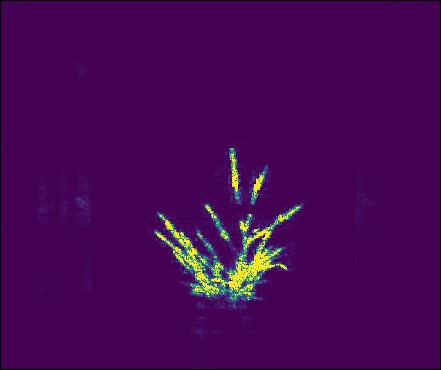


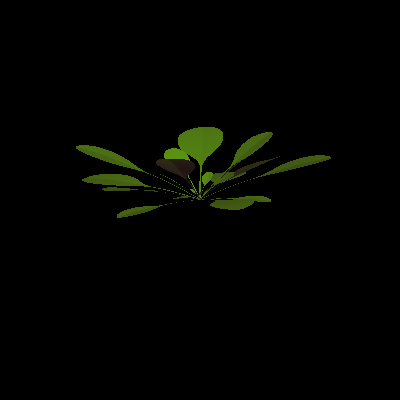

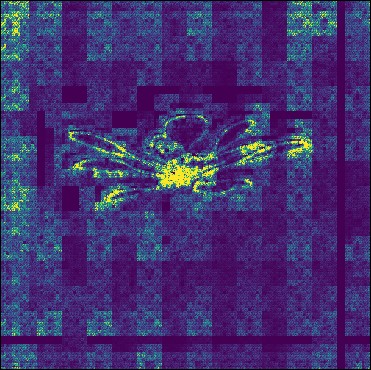


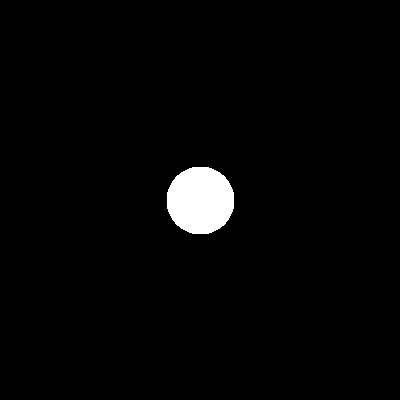

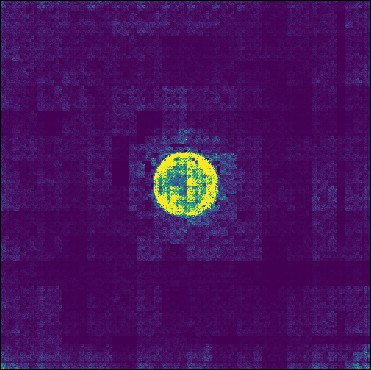


Figure S9: Example images from the *Setaria*, synthetic Arabidopsis, and synthetic circles datasets (left) and corresponding saliency maps generated using guided backpropagation (right). Intensity is higher for the pixels which have high saliency with respect to the latent space embedding of the image. The *Setaria* image is from an experiment carried out without cropping to include the background in the saliency demonstration.
